# Letter to the Editor: Venezuelan Equine Encephalitis virus 1B Invasion and Epidemic Control—South Texas, 1971

**DOI:** 10.3390/tropicalmed5020104

**Published:** 2020-06-22

**Authors:** Robert G. McLean

**Affiliations:** Retired, Research Program Manager, Wildlife Diseases Program, National Wildlife Research Center, Wildlife Services, USDA, APHIS, Fort Collins, CO 80521, USA; robertmclean285@gmail.com; Tel.: +01-970-391-1546

**Keywords:** Venezuelan equine encephalitis virus, outbreak, arbovirus, interagency, response, coordination, mosquito, vaccine, emerging virus

## Abstract

The epidemic strain of Venezuelan equine encephalitis virus (VEE) 1B invaded south Texas in 1971. The success of the eventual containment and control of the virus invasion was the early recognition and immediate detection, cooperation, coordination, and participation among multiple federal agencies. There were 4739 wild vertebrate animals trapped on a ranch in the area with only 1 VEE virus isolation from a Virgina opossum (Didelphis virginiana). A large number of mosquitoes were also collected on the ranch and tested, resulting in 240 VEE virus isolations. Virus isolations were obtained from 58% of the 33 equines tested. Wild vertebrates did not play a significant role in the outbreak.

## 1. Editor’s Preface

In the letter below, Dr. McLean provides a first-hand account of the invasion of Venezuelan equine encephalitis virus 1B into South Texas. This invasion was met with a massive, coordinated, inter-agency response, which ultimately ended the outbreak. We believe this event represents the sole example to date in which invasion of an exotic zoonotic arbovirus into the United States has been eradicated, and exemplifies the interagency cooperation necessary to achieve such a successful outcome. During a time where we can expect the continued emergence of viruses that threaten human and animal health, such lessons from the field can be both informative and encouraging. Dr. McLean has led a very distinguished career, serving in leadership positions in both the Center for Disease Control (CDC) and the United States Department Agriculture (USDA), and we value his insight and perspective on this historic outbreak.

### Dear Editor

The epidemic strain of Venezuelan equine encephalitis (VEE) 1B was responsible for epidemics in humans and equines in Ecuador in January 1969, and was subsequently introduced into coastal Guatemala and El Salvador (possibly in infected equines) in the summer of 1969. From there, the virus spread south to Honduras and Nicaragua, and north to Southern Mexico by November 1969. In 1970, the virus spread throughout those countries and north along the Gulf coast of Mexico toward Tampico, where it resulted in an epidemic there in March–April 1971. Descriptions of this outbreak have been published [1,2,3,4].

This continued northward progression alerted USDA Animal Plant Health Inspection Service (APHIS) and CDC to the likelihood of an invasion into the U.S. This impending hazard to the U.S. initiated the two agencies to start working together to develop contingency plans to address the hazard. A number of meetings were held among their staff and invited experts from other federal and state agencies and universities to develop strategies for prevention and control as well as to determine what investigations were needed. The U.S. Medical Research Institute of Infectious Diseases already had a VEE vaccine in their stock but it was not FDA/APHIS approved. However, it was made available to the team of investigators if they signed a voluntary release to protect them in Texas from VEE-1B virus infections that are quite debilitating in humans and kills equines. We were all willing to receive this unapproved (by FDA) vaccine so we could perform our duties in Texas. APHIS mobilized a number of teams of veterinarians, technicians, and administrators; CDC mobilized physicians, epidemiologists, entomologists (Dr. Vern Newhouse as leader), virologists (Dr. Charles Calisher as leader), wildlife biologists (Dr. Robert McLean as leader), and technicians for each. Counterpart staff from public health and other state agencies in Texas joined in the effort.

APHIS and CDC administrators and senior staff met in south Texas to develop local plans and establish administrative facilities and local and state connections and coordination. They were waiting for the first confirmed identification of VEE-1B virus in Texas (USA), whether equine, humans, or possibly mosquitoes or vertebrate animals. They could not enter Mexico to sample or test animals or humans to confirm the virus presence in nearby Northeast Mexico (across the Rio Grande river), and had to wait for it to spread into Texas. Arrangements were made to secure enough hotel and working space for the team members. All of the space at a motel in south Texas that was convenient for the potential affected area was reserved, and all of us later stayed there. We also used rooms to contain refrigerators and freezers, store equipment and supplies, setup a laboratory, and have meetings. It was the local epidemic investigation headquarters. APHIS had a large number of veterinarians there to deal with the investigation and control of VEE-1B virus in the equine population.

The team leaders selected staff to help conduct investigations, collected equipment and supplies and arranged for shipment to Southeast Texas, reserved vehicles and hotel reservations, and waited for the orders to proceed after the first VEE-1B virus detection in south Texas near the U.S.-Mexico border was confirmed. The first detection was a sick equine near the border. All of the teams were quickly mobilized to South Texas.

APHIS brought multiple teams of veterinarians for field investigations of affected equines and to establish quarantine and equine movement restrictions. CDC quickly setup a laboratory in the hotel to process and test blood samples for rapid detection of virus and antibody for infected equines and later humans after CDC epidemiologists began investigating human cases. All field supplies for all investigations of equine, human, mosquitoes, and wildlife were stored and distributed from that hotel, which was the headquarters for all of the entire U.S. government effort in South Texas. Coordination with state and local officials involved in the outbreak investigations was conducted, as well as with the press, from there.

Mosquito control efforts were developed, organized, and conducted locally, then broadened as the epidemic zone expanded. Finally, the U.S. Air Force fleet of large insecticide spray aircraft were deployed as the epidemic spread widely threatening larger areas of Texas to the north [5].

Because equines were the primary host for amplification, a big achievement for the control of the epidemic and the protection of equines was the very rapid emergency approval of the VEE vaccine by USDA and the production of the military VEE vaccine for use in equines by a private vaccine company. The vaccine was made available for use by private veterinarians throughout the state of Texas. One industrious veterinarian acquired state approval and distributed the vaccine by a rented private plane to veterinarians throughout the state. Control of the epidemic and advancement northward was achieved, but there were about 1800 equine cases in the southern half of the state by the time the epidemic was stopped more than two months later.

## 2. Wildlife Investigations

At this time, I was the only wildlife biologist/epidemiologist at CDC and was in the Rabies branch of the epidemiology program. I was requested and agreed to head the wildlife investigations and selected some team members to assist. Luckily, I had just hired a biologist from south Texas who had worked for CDC at the public health unit at the border there that was closing and who grew up in that area. He had many contacts there and helped arrange in selecting a sampling location near the border on a large cattle ranch with great habitats for wildlife. The rancher allowed us free access to all of the ranch and provided help and guidance from his ranch manager.

We set a large number of traps for rodents; smaller numbers of traps for larger mammals such as raccoons, opossums, and rabbits; and leg-hold traps for carnivores and captured tortoises by hand throughout the ranch. At the same time, we set up mist nets for birds throughout the area and operated some mist nets at night for bats. Canon nets were set up on the beach on South Padre Island to capture sea gulls. Mosquito light traps were placed within the lines of mist nets and mammal traps to obtain a comprehensive sampling of vector and vertebrate populations for a more thorough effort to detect the presence of VEE 1B virus. Entomologist Dr. Newhouse from CDC was the leader of the mosquito sampling effort, although we all worked together to conduct prolonged and intense field investigations during a multi-week period in August—September 1971.

Follow-up investigations were conducted multiple times at these sampling sites by the vertebrate and vector teams during the next year to ensure that VEE 1B virus did not become established in South Texas.

## 3. Laboratory Testing

Some of the testing of blood specimens from vertebrate animals, humans and processed mosquitoes were conducted locally to obtain rapid results for the epidemiologists and mosquito control teams who were trying to stop the epidemic expansion.

The majority of the testing was conducted at CDC in Atlanta. Virus isolation was performed by inoculating 0.02 mL of each sample by the intracerebral route into Swiss albino suckling mice. Virus isolates were identified by microtiter complement fixation tests. Virus neutralizing antibody in the vertebrate and human sera were tested in duck embryo cell culture by the plaque reduction neutralization test using VEE-TC-83 vaccine virus.

## 4. Conclusion

This was a massive, coordinated effort to prepare for the impending invasion of the epidemic 1B strain of VEE virus into the USA at the Texas border. The success of the eventual containment and control of the virus invasion was the early recognition and immediate detection, cooperation, coordination, and participation among multiple federal agencies of USDA, CDC, U.S. Military, National Park Service, Department of Interior), multiple Texas state departments, and local agencies and their staff with valuable input from individual scientific experts. The leaders of these agencies were on the ground in South Texas to initiate this massive, coordinated federal/state response. A well-planned response was developed, and multiple teams were formed to investigate specific health aspects in the human and equine populations, and the mosquito vector and wild vertebrate host involvement. The effective control of virus transmission was initiated quickly through broad equine vaccination and quarantine and intensive mosquito control that prevented the epidemic spread to Northern Texas and surrounding states. The close cooperation and joint leadership among the federal, state, and local officials and the dedicated and exhaustive efforts by the scientists and public health officials and their staff was key for the success of this dramatic disease control effort. It was a good example for dealing with foreign disease invasion and control. A big advantage for the control of this invasion and impending epidemic in Texas was the prior knowledge about the virus strain, history of its origin, and movement from South America to Central America and northward along the Gulf coast of Mexico, and the information on the potential domestic and wild vertebrate hosts and vectors that would be affected.
